# Supplementary material for: Ion occupancy of the selectivity filter controls opening of a cytoplasmic gate in the K2P channel TALK-2
Source: Nat Commun. 2024 Aug 30;15:7545. doi: 10.1038/s41467-024-51812-w (PMC11364775; doi:10.1038/s41467-024-51812-w)
Supplement: Supplementary file 1 — Supplementary Information [file 41467_2024_51812_MOESM1_ESM.pdf]

**Supplementary Information**

# Ion occupancy of the selectivity filter controls opening of a cytoplasmic gate in the $K_{2P}$ channel TALK-2

**Lea C. Neelsen[1], Elena B. Riel[1,2], Susanne Rinné[3], Freya-Rebecca Schmid[2], Björn C. Jürs[1,4], Mauricio Bedoya[5,6], Jan P. Langer[1], Bisher Eymsh[1], Aytug K. Kiper[3], Sönke Cordeiro[1], Niels Decher[3], Thomas Baukrowitz[1] & Marcus Schewe[1]**

[1]Institute of Physiology, Christian-Albrechts University of Kiel, Kiel, Germany
[2]Department of Anesthesiology, Weill Cornell Medical College, New York, USA
[3]Institute of Physiology and Pathophysiology, Philipps-University of Marburg, Marburg, Germany
[4]MSH Medical School Hamburg, University of Applied Sciences and Medical University, Hamburg, Germany
[5]Centro de Investigación de Estudios Avanzados del Maule (CIEAM), Vicerrectoría de Investigación y Postgrado, Universidad Católica del Maule, Talca, Chile
[6]Laboratorio de Bioinformática y Química Computacional (LBQC), Departamento de Medicina Traslacional, Facultad de Medicina, Universidad Católica del Maule, Talca, Chile

These authors contributed equally: Lea C. Neelsen, Elena B. Riel, Susanne Rinné
These authors jointly supervised this work: Niels Decher, Thomas Baukrowitz, Marcus Schewe
decher@staff.uni-marburg.de; t.baukrowitz@physiologie.uni-kiel.de; m.schewe@physiologie.uni-kiel.de

Supplementary Figures 1 - 7
Supplementary Tables 1 - 4

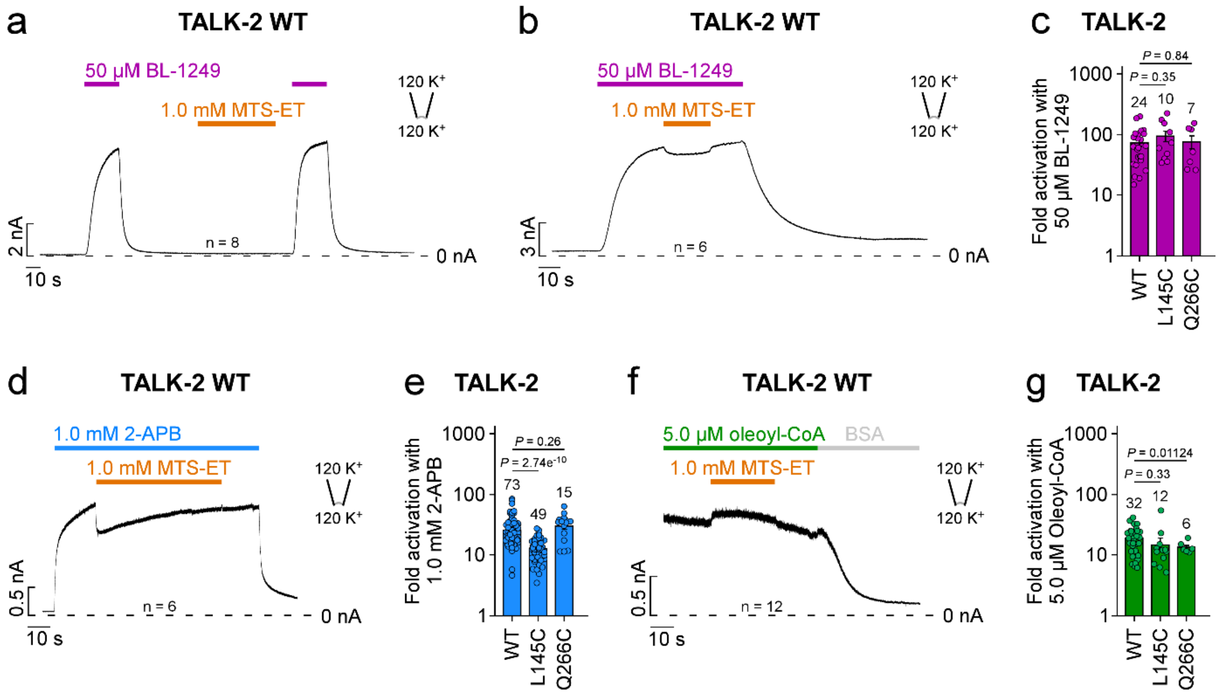


**Supplementary Figure 1 | Characterization of WT and mutant TALK-2 K$_{2P}$ channels for the cysteine modification assay. a**, **b** Representative measurement of WT TALK-2 channels under voltage-clamp conditions at +40 mV from an inside-out patch of a oocyte using symmetrical $K^+$ concentrations (120 mM $[K^+]_{ex.}$/120 mM $[K^+]_{int.}$) at pH 7.4 showing the application of 1.0 mM MTS-ET for 25 - 40 s to the intracellular side of the membrane in the basal state (a) and in the BL-1249-activated state (b). Note, TALK-2 channel expression was verified by application of 50 µM BL-1249 resulting in robust and reversible channel activation. **c** Fold activation analyzed at +40 mV of WT and mutant (L145C and Q266C) TALK-2 channel currents with intracellular applied 50 µM BL-1249. **d-g** Representative WT TALK-2 channel currents measured as in (b) activated with either 1.0 mM 2-APB (d) or 5.0 µM oleoyl-CoA (f) showing almost no effect of intracellular applied 1.0 mM MTS-ET within 30 - 40 s. Note, oleoyl-CoA washout was accelerated with an intracellular bath solution containing bovine serum albumin (BSA; 5 mg/ml). Fold activation analyzed at +40 mV of WT and mutant (L145C and Q266C) TALK-2 channel currents with intracellular applied 1.0 mM 2-APB (e) or 5.0 µM oleoyl-CoA (g). Data shown are the mean ± s.e.m and the number (n) of independent experiments is indicated above the bars and repeats of representative experiments with the similar results in the figure. Statistical relevance has been evaluated using unpaired, two-sided *t*-test and exact *P* values are indicated in the figure.

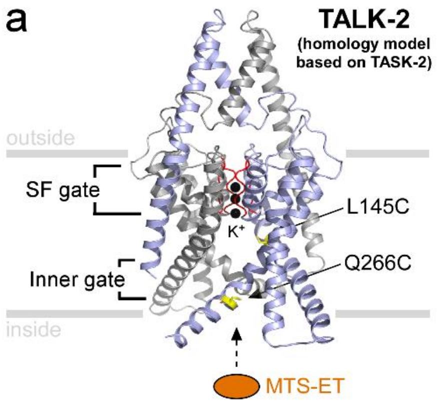


**Supplementary Figure 2 | Homology model of TALK-2 based on TASK-2. a** Pore homology model of TALK-2 based on the cryo-EM structure of closed TASK-2 at pH 6.5 (PDB ID: 6WLV) with the SF highlighted in red, $K^+$ ions in black and cysteine residues (L145C and Q266C) for MTS-ET modification in yellow.

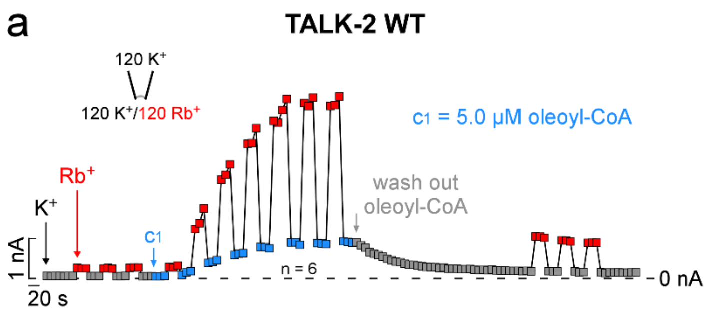


**Supplementary Figure 3 | Probing the status of the lower gate in TALK-2 with oleoyl-CoA. a** Analysis of WT TALK-2 channel currents at +40 mV from voltage ramps measured in an inside-out patch and symmetrical ion conditions (120 mM $[K^+]_{ex.}$/120 mM $[X^+]_{int.}$) showing channel activation with 5.0 µM oleoyl-CoA (blue squares). Note, 5.0 µM oleoyl-CoA activation occurs with a slow time course facilitating direct $K^+$/$Rb^+$ exchange in the process of wash in (red squares). The activatory effect of $Rb^+$ increased with rising oleoyl-CoA activation. The experiment was repeated with similar results (n = 6).

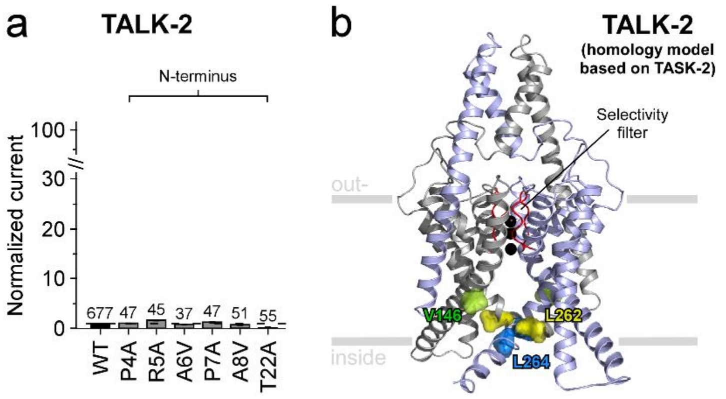


**Supplementary Figure 4 | Systematic alanine screening and mapping of TALK-2 K2P channel mutants. a** Relative current amplitudes from two-electrode voltage-clamp (TEVC) measurements at pH 8.5 of WT and mutant TALK-2 channels 24 h after injection of 25 ng cRNA in oocytes. Currents were elucidated with a voltage protocol ramped from -120 mV to +45 mV within 3.5 s, analyzed at +40 mV and normalized to WT. **b** Pore homology model of TALK-2 based on the cryo-EM structure of closed TASK-2 at pH 6.5 (PDB ID: 6WLV, chains A, B) highlighting the cluster of g-o-f mutations (V146A, L262A and L264A) at the cytosolic pore entrance. Data shown are the mean ± s.e.m and the number (n) of independent experiments is indicated above the bars in the figure.

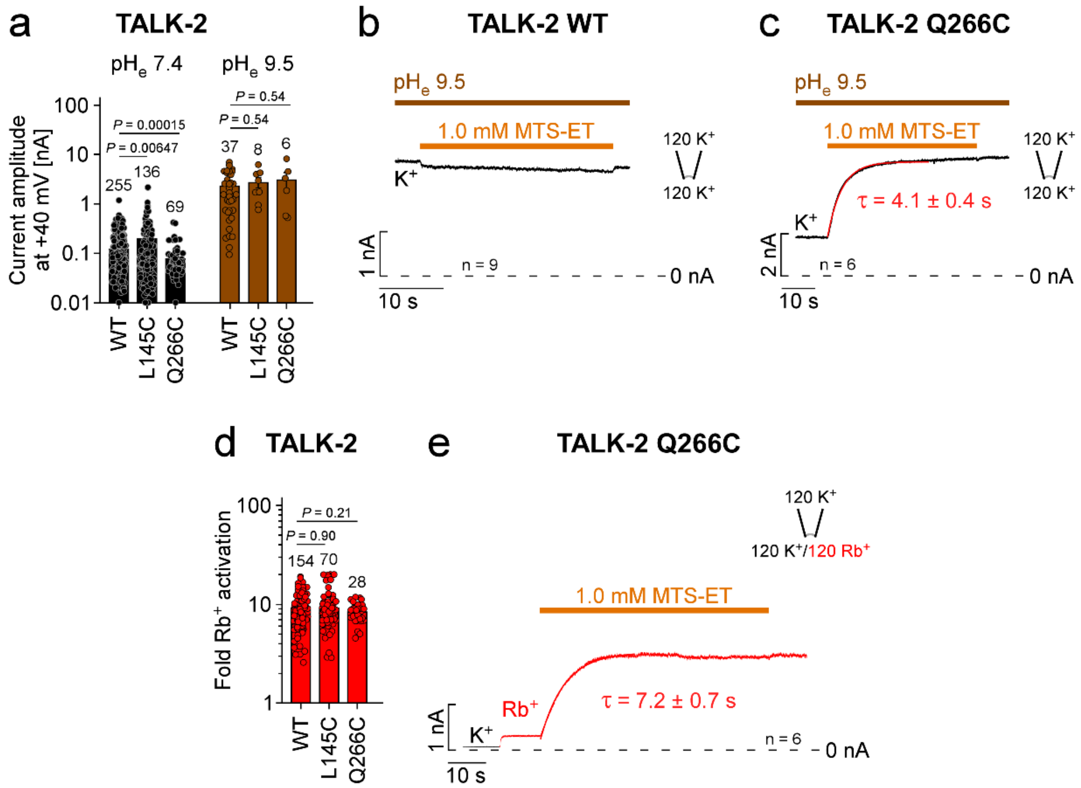


**Supplementary Figure 5 | Validation of the cysteine modification assay with $Rb^+$ as permeant ion and extracellular alkalinization. a** Current amplitudes analyzed at +40 mV of WT and mutant (L145C and Q266C) TALK-2 channels measured in inside-out patches of oocytes using symmetrical $K^+$ concentrations (120 mM $[K^+]_{ex.}$/120 mM $[K^+]_{int.}$) at $pH_e$ 7.4 (black bars) and $pH_e$ 9.5 (brown bars), respectively. **b**, **c** Representative measurements of WT (b) and Q266C mutant TALK-2 channels (c) at +40 mV pre-activated by extracellular alkalinization ($pH_e$ 9.5) showing the effect of 1.0 mM MTS-ET applied to the intracellular side of the membrane. **d** Fold activation of WT and mutant (L145C and Q266C) TALK-2 channel currents at +40 mV with intracellular $Rb^+$. **e** Representative measurement of TALK-2 Q266C mutant channels with 1.0 mM MTS-ET applied for 60 s at +40 mV in the $Rb^+$-activated state. Data shown are the mean ± s.e.m and the number (n) of independent experiments is indicated above the bars and repeats of representative experiments with the similar results in the figure. Statistical relevance has been evaluated using unpaired, two-sided *t*-test and exact *P* values are indicated in the figure.

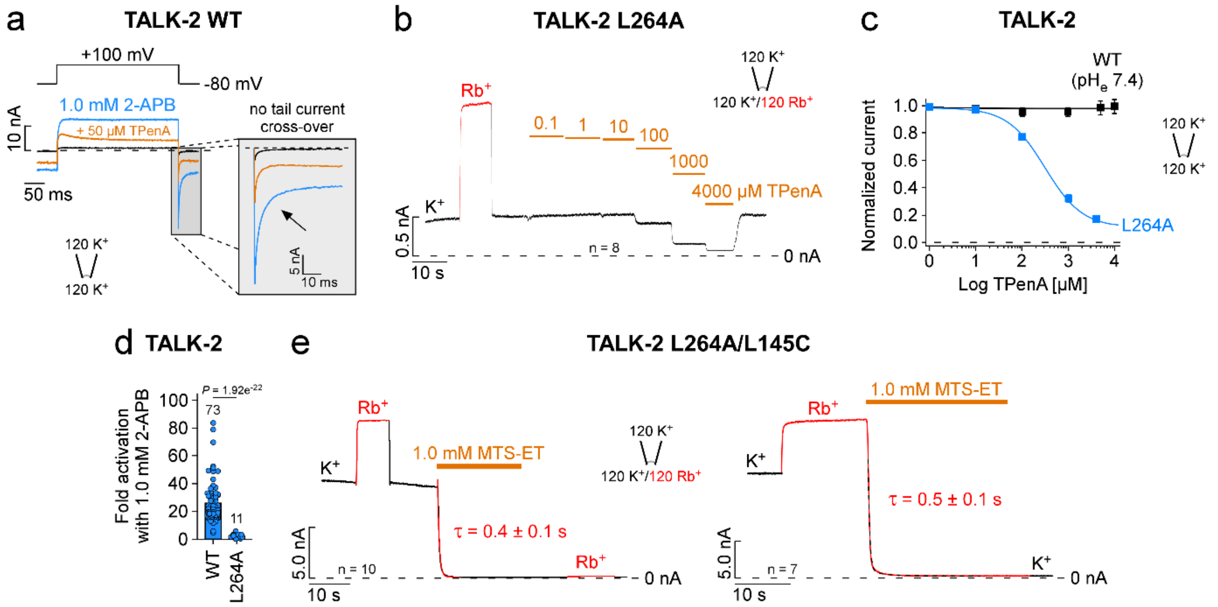


**Supplementary Figure 6 | Characteristics of pore block and cysteine modification in L264A TALK-2 $K_{2P}$ channels. a** Current responses measured in an inside-out patch expressing WT TALK-2 channels activated with the indicated voltage step under symmetrical ion conditions at pH 7.4 with either intracellular $K^+$ (black trace, basal state) or 1.0 mM 2-APB (blue trace, activated state) and with 1.0 mM TPenA in 2-APB (orange trace). Note, TPenA has no effect on the tail current deactivation kinetics. **b** Representative measurement of TALK-2 L264A channels at +40 mV in an excised membrane patch in symmetrical $K^+$ at pH 7.4 showing a dose-dependent TPenA inhibition with the indicated concentrations applied from the intracellular side to the membrane. Notably, the basal $K^+$ currents of the mutant TALK-2 channels display a g-o-f phenotype in respect to WT TALK-2 channels, however a reduced $Rb^+$ sensitivity is retained in comparison to WT. **c** Dose-response curves of TPenA inhibition from measurements as in (b) for WT TALK-2 channels in unstimulated (basal) conditions (black) and for L264A mutant channels (blue) with a TPenA $IC_{50}$ of 459 ± 58 µM. **d** Fold activation of WT and L264A mutant TALK-2 channel currents with 1.0 mM 2-APB. **e** (left) Representative measurement of TALK-2 L264A mutant channels additionally carrying the inner pore mutation L145C (TALK-2 L264A/L145C) at +40 mV from an excised patch under symmetrical ion conditions at pH 7.4 showing a fast and irreversible modification upon application of 1.0 mM MTS-ET (τ = 0.4 ± 0.1 s). Notably, the TALK-2 double mutant (L264A/L145C) can be activated with intracellular $Rb^+$ before, but not after chemical modification that subsequently blocks the permeation pathway. **e** (right) Modification of TALK-2 L264A/L145C channels in the $Rb^+$-activated

state showing the same time course (τ = 0.5 ± 0.1 s). Data shown are the mean ± s.e.m and the number (n) of independent experiments is indicated above the bars and repeats of representative experiments with the similar results in the figure. Statistical relevance has been evaluated using unpaired, two-sided *t*-test and exact *P* values are indicated in the figure.

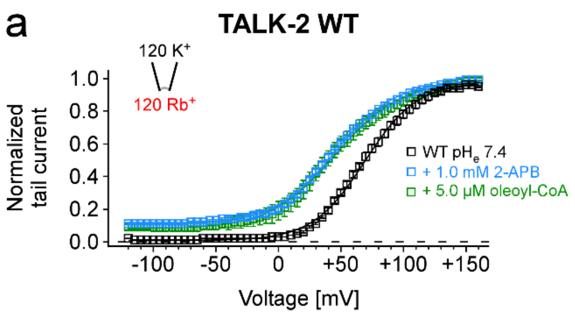


**Supplementary Figure 7 | Activation of WT and mutant TALK-2 $K_{2P}$ channels with alkaline $pH_e$, 2-APB and oleoyl-CoA. a** G-V curves analyzed from tail currents at -80 mV after 300 ms pre-pulses (-120 mV to +160 mV with 5 mV increments) under symmetrical ion conditions with intracellular $Rb^+$ for unstimulated (black trace) and pre-activated WT TALK-2 channels with either 1.0 mM 2-APB (blue trace) or 5.0 µM oleoyl-CoA (green trace). **b** Normalized current from TEVC measurements of oocytes expressing WT (n = 11) and mutant L262A (n = 5) or L264A TALK-2 channels (n = 8), respectively. Channels were activated by increasing $pH_e$ from 5.5 to 10.5 with 0.5 pH increments. Currents were elucidated with a voltage protocol ramped from -120 mV to +45 mV within 3.5 s, analyzed at +40 mV and normalized to pH 8.5 for the respective channel. Data shown are the mean ± s.e.m and number (n) of independent experiments is indicated in supplementary table 4.

**Supplementary Table 1 | Chemical modification of WT and mutant TALK-2 (TASK-4) channels with MTS-ET.** Time course and modification rate with 1 mM MTS-ET at +40 mV for TALK-2 (TASK-4) channels in a symmetrical ion gradient at different states as indicated. Data are presented as mean ± s.e.m. with the number (n) of independent experiments.

| $K_{2P}$ channel | Mutation | State | τ of MTS-ET modification [s] | Modification rate [1/s] | n |
|---|---|---|---|---|---|
| **hTALK-2** | WT | basal ($K^+$ $pH_i$ 7.4) | no modification (within 60 s) | - | 8 |
| **hTALK-2** | WT | activated ($Rb^+$ $pH_i$ 7.4) | no modification (within 60 s) | - | 8 |
| **hTALK-2** | WT | activated (1.0 mM 2-APB) | no modification (within 60 s) | - | 6 |
| **hTALK-2** | WT | activated (5.0 µM Oleoyl-CoA) | no modification (within 30 s) | - | 12 |
| **hTALK-2** | WT | activated (50 µM BL-1249) | no modification (within 30 s) | - | 6 |
| **hTALK-2** | WT | activated ($K^+$ $pH_e$ 9.5) | no modification (within 30 s) | - | 9 |
| **hTALK-2** | L145C | basal ($K^+$ $pH_i$ 7.4) | no modification (within 30 s) | - | 14 |
| **hTALK-2** | L145C | activated ($Rb^+$ $pH_i$ 7.4; +20 mV) | 46.3 ± 6.4 | 0.03 ± 0.00 | 12 |
| **hTALK-2** | L145C | activated ($Rb^+$ $pH_i$ 7.4; +40 mV) | 16.8 ± 1.9 | 0.07 ± 0.01 | 20 |
| **hTALK-2** | L145C | activated ($Rb^+$ $pH_i$ 7.4; +80 mV) | 5.7 ± 0.8 | 0.24 ± 0.04 | 15 |
| **hTALK-2** | L145C | activated ($Rb^+$ $pH_i$ 7.4; +115 mV) | 4.3 ± 0.6 | 0.26 ± 0.04 | 6 |
| **hTALK-2** | L145C | activated ($Rb^+$ $pH_i$ 7.4; +140 mV) | 3.8 ± 0.8 | 0.31 ± 0.05 | 7 |
| **hTALK-2** | L145C | activated (0.01 mM 2-APB) | 36.5 ± 6.2 | 0.03 ± 0.00 | 9 |
| **hTALK-2** | L145C | activated (0.1 mM 2-APB) | 24.3 ± 2.5 | 0.05 ± 0.01 | 15 |
| **hTALK-2** | L145C | activated (0.2 mM 2-APB) | 19.2 ± 5.9 | 0.06 ± 0.02 | 3 |
| **hTALK-2** | L145C | activated (0.5 mM 2-APB) | 11.9 ± 1.8 | 0.11 ± 0.02 | 11 |
| **hTALK-2** | L145C | activated (1.0 mM 2-APB) | 12.8 ± 0.7 | 0.08 ± 0.01 | 9 |
| **hTALK-2** | L145C | activated (2.0 mM 2-APB) | 10.8 ± 1.4 | 0.10 ± 0.01 | 8 |
| **hTALK-2** | L145C | activated (5.0 µM Oleoyl-CoA) | 13.5 ± 2.1 | 0.09 ± 0.02 | 7 |
| **hTALK-2** | L145C | activated ($K^+$ $pH_e$ 9.5) | 12.5 ± 1.8 | 0.09 ± 0.01 | 8 |

**Supplementary Table 2 | Chemical modification of WT and mutant TALK-2 (TASK-4) channels with MTS-ET.** Time course and modification rate with 1 mM MTS-ET at +40 mV for TALK-2 (TASK-4) channels in a symmetrical ion gradient at different states as indicated. Data are presented as mean ± s.e.m. with the number (n) of independent experiments.

| $K_{2P}$ channel | Mutation | State | τ of MTS-ET modification [s] | Modification rate [1/s] | n |
|---|---|---|---|---|---|
| **hTALK-2** **(TASK-4)** | L264A/ L145C | activated (L264A in $K^+$ $pH_i$ 7.4; gain-of-function mutation) | 0.4 ± 0.1 | 2.45 ± 0.29 | 10 |
| **hTALK-2** **(TASK-4)** | L264A/ L145C | activated (L264A in $Rb^+$ $pH_i$ 7.4; gain-of-function mutation) | 0.5 ± 0.1 | 2.84 ± 0.46 | 7 |
| **hTALK-2** **(TASK-4)** | V146A/ L145C | activated (V146A in $K^+$ $pH_i$ 7.4; gain-of-function mutation) | 4.9 ± 0.7 | 0.24 ± 0.03 | 10 |
| **hTALK-2** **(TASK-4)** | L262A/ L145C | activated (L262A in $K^+$ $pH_i$ 7.4; gain-of-function mutation) | 7.8 ± 0.9 | 0.14 ± 0.01 | 7 |
| **hTALK-2** **(TASK-4)** | W255A/ L145C | activated (W255A in $K^+$ $pH_i$ 7.4; gain-of-function mutation) | 11.5 ± 3.2 | 0.14 ± 0.03 | 8 |
| **hTALK-2** | Q266C | basal ($K^+$ $pH_i$ 7.4) | 7.1 ± 0.7 | 0.16 ± 0.02 | 9 |
| **hTALK-2** | Q266C | activated ($Rb^+$ $pH_i$ 7.4) | 7.2 ± 0.7 | 0.15 ± 0.01 | 6 |
| **hTALK-2** | Q266C | activated (1.0 mM 2-APB) | 7.1 ± 0.8 | 0.17 ± 0.04 | 8 |
| **hTALK-2** | Q266C | activated (5.0 μM Oleoyl-CoA) | 11.5 ± 2.0 | 0.10 ± 0.02 | 6 |
| **hTALK-2** | Q266C | activated ($K^+$ $pH_e$ 9.5) | 4.1 ± 0.4 | 0.26 ± 0.03 | 6 |

**Supplementary Table 3 | Parameters of voltage activation.** $V_{1/2}$ and z values of WT and mutant TALK-2 (TASK-4) channels from Boltzmann fitted G-V curves with intracellular $Rb^+$. Data are presented as mean ± s.e.m. with the number (n) of independent experiments. n. e. = no expression, n. d. = not determinable.

| $K_{2P}$ channel | Mutation | TM region | $V_{1/2}$ (mV) | z | n |
|---|---|---|---|---|---|
| **hTALK-2** (hTASK-4) | WT | - | 71.6 ± 2.4 | 1.21 ± 0.06 | 15 |
| **hTALK-2** | V139A | TM2 | 64.0 ± 6.5 | 1.06 ± 0.05 | 7 |
| **hTALK-2** | N144A | TM2 | n. e. | n. e. | - |
| **hTALK-2** | L145A | TM2 | 61.6 ± 3.9 | 1.12 ± 0.04 | 13 |
| **hTALK-2** | L145C | TM2 | 65.8 ± 2.6 | 0.97 ± 0.03 | 10 |
| **hTALK-2** | V146A | TM2 | 29.6 ± 1.9 | 1.54 ± 0.13 | 6 |
| **hTALK-2** | V147A | TM2 | 83.3 ± 4.7 | 0.92 ± 0.04 | 3 |
| **hTALK-2** | Q157A | TM2 | 65.1 ± 4.1 | 1.11 ± 0.07 | 6 |
| **hTALK-2** | G252A | TM4 | n. e. | n. e. | - |
| **hTALK-2** | M253A | TM4 | 38.8 ± 1.9 | 1.24 ± 0.19 | 6 |
| **hTALK-2** | A254V | TM4 | n. d. | n. d. | 9 |
| **hTALK-2** | W255A | TM4 | 30.3 ± 1.4 | 1.68 ± 0.10 | 14 |
| **hTALK-2** | L256A | TM4 | 75.8 ± 4.1 | 0.96 ± 0.03 | 6 |
| **hTALK-2** | A257V | TM4 | n. d. | n. d. | 10 |
| **hTALK-2** | L258A | TM4 | 54.7 ± 2.8 | 1.39 ± 0.10 | 6 |
| **hTALK-2** | I259A | TM4 | 84.5 ± 3.6 | 1.02 ± 0.06 | 12 |
| **hTALK-2** | I260A | TM4 | 88.7 ± 3.7 | 0.69 ± 0.04 | 7 |
| **hTALK-2** | K261A | TM4 | 76.2 ± 3.2 | 1.10 ± 0.04 | 7 |
| **hTALK-2** | L262A | TM4 | 11.7 ± 4.1 | 1.51 ± 0.14 | 8 |
| **hTALK-2** | I263A | TM4 | 71.4 ± 3.5 | 0.96 ± 0.03 | 9 |
| **hTALK-2** | L264A | TM4 | -12.4 ± 2.9 | 1.21 ± 0.13 | 15 |
| **hTALK-2** | L264A (+ 1.0 mM TPenA) | TM4 | 8.7 ± 3.5 | 1.53 ± 0.11 | 8 |
| **hTALK-2** | S265A | TM4 | 71.4 ± 2.7 | 1.01 ± 0.03 | 10 |
| **hTALK-2** | Q266A | TM4 | 81.0 ± 2.6 | 0.97 ± 0.03 | 6 |
| **hTALK-2** | L267A | TM4 | 96.4 ± 2.9 | 0.85 ± 0.04 | 6 |
| **hTALK-2** | E268A | TM4 | n. d. | n. d. | 6 |

**Supplementary Table 4 | Parameters of voltage activation.** $V_{1/2}$ and z values of WT TALK-2 (TASK-4) channels from Boltzmann fitted G-V curves with intracellular $Rb^{+}$ and indicated stimuli. Data are presented as mean ± s.e.m. with the number (n) of independent experiments.

| $K_{2P}$ channel | Stimuli | TM region | V1/2 (mV) | z | n |
|---|---|---|---|---|---|
| **hTALK-2** **(hTASK-4)** | WT ($pH_e$ 7.4) | - | 71.6 ± 2.4 | 1.21 ± 0.06 | 15 |
| **hTALK-2** | WT (+ 1.0 mM TPenA) | - | 25.5 ± 2.5 | 1.74 ± 0.04 | 8 |
| **hTALK-2** | WT (+ 1.0 mM 2-APB) | - | 44.7 ± 1.1 | 1.08 ± 0.07 | 5 |
| **hTALK-2** | WT (+ 5.0 µM Oleoyl-CoA) | - | 44.9 ± 5.4 | 1.04 ± 0.11 | 7 |
| **hTALK-2** | WT ($pH_e$ 7.0) | - | 73.9 ± 2.2 | 1.10 ± 0.05 | 6 |
| **hTALK-2** | WT ($pH_e$ 8.0) | - | 63.2 ± 1.2 | 1.1 ± 0.05 | 6 |
| **hTALK-2** | WT ($pH_e$ 8.5) | - | 47.0 ± 3.7 | 1.27 ± 0.10 | 7 |
| **hTALK-2** | WT ($pH_e$ 9.0) | - | 38.0 ± 1.5 | 1.51 ± 0.20 | 6 |
| **hTALK-2** | WT ($pH_e$ 9.5) | - | 28.8 ± 2.6 | 0.93 ± 0.05 | 9 |
| **hTALK-2** | WT ($pH_e$ 10.0) | - | 30.1 ± 3.5 | 1.06 ± 0.15 | 9 |
| **hTALK-2** | WT ($pH_e$ 10.5) | - | 21.8 ± 2.8 | 1.49 ± 0.17 | 6 |
| **hTREK-2** | WT ($pH_e$ 7.4) | - | 34.6 ± 2.5 | 2.12 ± 0.10 | 6 |
| **hTREK-2** | WT (+ 0.1 mM TPenA) | - | 27.2 ± 2.4 | 2.01 ± 0.14 | 7 |
| **hTREK-2** | WT (+ 1.0 mM TPenA) | - | 32.0 ± 1.4 | 1.52 ± 0.08 | 7 |
